# The intensity of horizontal and vertical search in a diving forager: the harbour seal

**DOI:** 10.1186/s40462-015-0042-9

**Published:** 2015-05-27

**Authors:** Virginie Ramasco, Frédéric Barraquand, Martin Biuw, Bernie McConnell, Kjell T Nilssen

**Affiliations:** Institute of Marine Research, Tromsø, Norway; University of Tromsø, Tromsø, Norway; Akvaplan-niva, Tromsø, Norway; Sea Mammal Research Unit, St. Andrews, UK

**Keywords:** Foraging index, Movement response, Benthic diving, Profitability, Predatory strategy, Resting

## Abstract

**Background:**

Free ranging foraging animals can vary their searching intensity in response to the profitability of the environment by modifying their movements. Marine diving animals forage in a three dimensional space and searching intensity can be varied in both the horizontal and vertical planes. Therefore understanding the relationship between the allocation of searching effort in these two spaces can provide a better understanding of searching strategies and a more robust identification of foraging behaviour from the multitude of foraging indices (FIs) available. We investigated the movement of a widespread marine coastal predator, the harbour seal (*Phoca vitulina*), and compared two sets of foraging indices reflecting searching intensity respectively in the horizontal plane (displacement speed, extensive vs. intensive movement types, residence time) and in the vertical dimension (time at the bottom of a dive). We then tested how several factors (dive depth, direction of the trip with respect to haul-out site, different predatory tactics, the presence of factors confounding the detection of foraging, and temporal resolution of the data) affected their relationships.

**Results:**

Overall the indices only showed a very weak positive correlation across the two spaces. However controlling for various factors strengthened the relationships. Resting at sea, a behaviour intrinsically static in the horizontal plane, was found to be strongly negatively related to the time spent at the bottom of the dives, indirectly weakening the relationship between horizontal and vertical foraging indices. Predatory tactic (benthic vs. pelagic) was found to directly affect the relationship. In benthic (as opposed to pelagic) foraging a stronger positive relationship was found between vertical and horizontal indices.

**Conclusions:**

Our results indicated that movement responses, leading to an intensification of search, are similar in the two spaces (positive relationship), but additional factors need to be taken into account for this relationship to emerge. Foraging indices measuring residence in the horizontal plane tend to be inflated by resting events at sea, while vertical indices tend to distinguish mainly between periods of activity and inactivity, or of benthic and pelagic foraging. The simultaneous consideration of horizontal and vertical movements, as well as topographic information, allows additional behavioural states to be inferred, providing greater insight into the interpretation of foraging activity.

**Electronic supplementary material:**

The online version of this article (doi:10.1186/s40462-015-0042-9) contains supplementary material, which is available to authorized users.

## Background

**Fig. 1:**
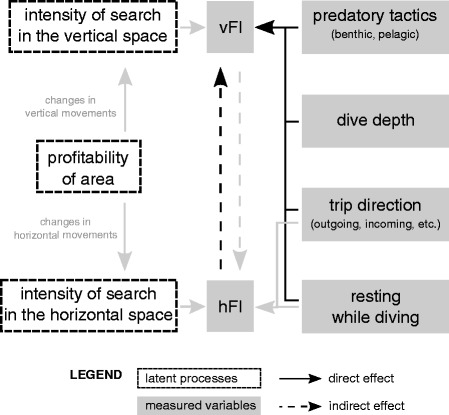
The factors affecting the relationship between the intensity of searching effort in the two spaces. The conceptual diagram shows latent and measured variables, together with their direct and indirect relationships (see legend). The assumed direct relationship between the intensity of search in the horizontal and vertical spaces, modulated by changes in movements in response to the area’s profitability, results in a potential indirect relationship between the derived horizontal and vertical foraging indices (hFIs, vFIs). The indices and their relationship may be affected by factors influencing the behaviour of the animals, such as predatory tactics, dive depth, trip direction and the presence of resting behaviour, or by methodological issues, such as the resolution of the data. The relationships between variables investigated in this study are represented by black arrows (not investigated = grey arrows)

The movement and time budgets of foraging animals are influenced by previously acquired knowledge and current assessment of resource distribution and quality [1, 2]. Optimal foraging models predict that, in order to achieve higher energetic gains, animals should spend more time in areas of increased profitability [3]. Free ranging animals can increase the time spent per unit area searching for food, hereafter called searching intensity, by switching from high movement speed and strong directionality (*i.e.* extensive movements) to lower speed and increased turning frequency (*i.e.* intensive movements) [4, 5] (see Fig. 1). The change between extensive to intensive movement patterns may therefore be used to infer the onset of foraging behaviour [6, 7]. However, the relationship between the inferred searching activity and the actual resource quality is not easy to estimate due to the difficulty in measuring the latter appropriately and the behavioural response to resources is therefore hard to measure. This is true in particular in environments difficult to access, such as marine habitats, for which resource quality is often inferred from physical proxies [8, 9].

Marine diving animals forage in a three dimensional space and searching intensity can be increased by modifying movements both in the horizontal plane and in the vertical dimension. Such changes can jointly provide valuable information about time allocation strategy while foraging [10]. Several studies on different marine diving species have compared indices derived from the animals’ use of space and intensity of their searching effort in the horizontal and vertical spaces, hereafter called foraging indices (FIs). In particular, these studies have tried to correlate foraging indices in the two spaces in order to provide a more robust identification of foraging behaviour [11–18]. Results across studies, even when performed on the same species, have led to different conclusions on the strength of this relationship. Bailleul et al. [11] for example found a lack of correlation in elephant seals, while Thums et al. [14] found a good mapping between horizontal movement and proxies of foraging success derived from dive data in the same species. Reasons for the temporal and spatial mismatch of horizontal and vertical indices (hFIs & vFIs) have been suggested to be mainly methodological, such as low spatial and temporal resolution of the data [12] or the lack of a unique association between the foraging indices and the behavioural states of interest (*e.g.* a given dive shape is not always associated to foraging success and vice versa, [15, 19, 20]).

The allocation of time in the different dimensions, in fact, is affected by different constraints. Dive time budgets are physiologically regulated by oxygen balance [21, 22], while horizontal movements in central place foraging pinnipeds are bound to regular return trips to the haul-out sites [23]. This suggests that even though profitability of an area can be expected to trigger changes in movements in both spaces, external factors and internal states, acting on an animal’s decision making [24], may confound any correlation.

Here we investigated the strategy of searching effort allocation between the horizontal and vertical spaces in harbour seals (*Phoca vitulina*). This species is a widespread coastal predator often targeting fishes of economic value [25, 26]. The ability to robustly identify harbour seals’ foraging behaviour is therefore of interest for estimating its role in the coastal environment and its potential impacts on relevant fish stocks. To characterize the intensity of search in the two spaces we considered two sets of foraging indices. For the horizontal plane we looked at horizontal displacement speed (HS), movement type (MT, extensive vs. intensive) and residence time (RT, [6]). Displacement speed is a variable extensively used in the analysis of horizontal movements in free ranging animals in different analytical frameworks (*i.e.* state-space models, [7, 27]); movement type is an output of switching state-space models [7] and integrates information on speed and turning frequency; residence time is an index, recently developed as a modification of First Passage Time [28], able to identify Area Restricted Search (ARS) behaviour by synthesizing the information on speed, turning angles and repetitive visits in one measure [6]. For the vertical dimension different numerical foraging indices have been employed in previous works, such as the Time at Depth index (TAD, [29]), or the bottom time (BT, *i.e.* the time spent at the bottom of a dive, [11, 17, 30]). These indices are based on the division of a foraging dive into travelling sections (*i.e.* descent and ascent) and a bottom phase at the depth of interest. The time at the bottom of a dive can therefore be considered a proxy of searching effort at depth [17].

We suggest that the relationship between horizontal and vertical foraging indices is likely to be influenced by factors such as the use of different predatory tactics, the main direction of movement between haul-out sites and feeding grounds, and dive depth (Fig. 1). In addition, methodological issues, such as the temporal resolution of the data and the presence of multiple behaviours showing similar movement signatures, may affect the ability of the indices in inferring foraging. Harbour seals feed mainly on gadoids, small pelagic fishes (*e.g.* herring), and bottom dwelling fishes, such as sandeel, and flatfish [31, 32]. In the area where the present study was located, large numbers of sculpins have been additionally reported in the diet [Institute of Marine Research, unpublished data]. Harbour seals can therefore be considered as both pelagic and benthic foragers and have been described using different predatory tactics depending on the prey type and its location in the water column, leading to different allocation of times in different phases of the dive [33]. Dive depth can also act as a potential constraint on the seal’s dive time budgets [21]. The duration of a dive’s bottom phase may therefore differ between benthic and pelagic dives and may depend on a dive’s maximum depth, generating variation in vertical foraging indices.

Outside of the reproductive season (June-August), harbour seals perform foraging trips returning regularly to haul-out sites mainly for resting [34]. Since feeding frequently occurs relatively close to the haul-out areas [35], it can be assumed that the animals have a higher degree of satiation when returning from foraging grounds then when leaving the haul-out sites. The two main directions of movement during a foraging trip (*i.e.* from and to the haul-out site) may therefore show differences in the intensity of search, potentially influencing the relationship between foraging indices. Harbour seals have been shown to rest, not only at haul-out sites, but also in the water, both at surface and while diving. These behaviours have been suggested to produce similar horizontal movement patterns as Area Restricted Search (ARS) behaviour depending on the temporal resolution of the tracks [36]. The presence of multiple behaviours with a similar horizontal movement signature may therefore be a potentially confounding factor in the relationship between searching intensities in the two spaces.

In this paper we consider four types of relationship between vertical and horizontal foraging indices, which may reflect different strategies of allocation of searching effort between the horizontal plane and the vertical dimension:

1. positive relationship: this suggests that changes in movements, leading to an increase in searching intensity in the two spaces, occur in response to a common latent cause (assumed increase in profitability);
2. positive relationship given conditioning factors: this suggests that there is a coherent movement response in the two spaces depending on external or internal factors;
3. negative relationship: this suggests that the allocation of search in the two planes may be used as alternative strategies;
4. no relationship: this suggests that changes in searching intensity in the different dimensions do not respond to a common latent cause.

The different outcomes have implications in the interpretation of the indices used for the detection of foraging based exclusively on either horizontal or vertical movements, as is often the case in many studies of aquatic animals. The validity of hypotheses three or four would jeopardize the robustness of the single indices in their ability to distinguish foraging from other behaviours, while the validity of hypothesis two implies that several conditioning factors may need to be taken into account for a better interpretation of the indices.

Our results supported hypothesis two). While differences in dive depth or in the main direction of movement during a foraging trip had a relatively small impact on the foraging indices and their relationship, the type of resource targeted and therefore the predatory tactic used (benthic vs. pelagic foraging) affected both searching intensity in the vertical dimension and its relationship with searching intensity in the horizontal plane. The confounding presence of multiple behaviours with a similar horizontal movement signature (*i.e.* resting while diving and foraging) may also strongly affect the interpretation of foraging indices based on searching intensity if not properly addressed.

## Results

A total of 14 harbour seals (four females, 10 males, all juveniles one-two years of age) were captured during the fall (August-October) of the years 2009-2012 [see Additional file 1]. The animals were tracked for on average 6.2 months (range 0.7 – 10.4), providing an average of 27.8 GPS positions (range 14.0 – 38.6) and 490 dives (range 212 – 642) per day per individual. The hFIs chosen for the comparison, horizontal displacement speed (HS), movement type (MT) and residence time (RT), represented an increasing degree of information integration. Since the theoretical expectation of bottom time was assumed to be positively affected by dive depth in a non-linear way [21], BT was standardized across depths and chosen as the vFI in the analysis. The derived index, standardized bottom time (stBT), was defined as the proportion of the maximum possible time, given a certain dive depth and duration, which is spent at the bottom of the dive, producing a value bound between zero and one [see Methods for definition]. This indicates that the closer the descending and ascending speeds are to the maximum observed vertical speed, the higher the stBT. This index can therefore be interpreted as a form of optimising time use at the maximum depth of the dive, which is assumed to be the depth of interest for the animal. When comparing bottom time to its standardized counterpart, a non-linear but positive relationship was found [see Additional file 2 for a correlation analysis between stBT and similar vFIs not used in the analysis], suggesting that higher efficiency in time use corresponds also to longer times spent at the dives’ bottom. Despite the standardization across depths however, standardized bottom time was still found to be partially correlated with dive depth (corr. = 0.35), which was therefore included as a conditioning factor in the models.

Switching state-space models [37] were fitted to estimate locations at regular time intervals (20 min) and associated movement types. Horizontal speed computed on the resulting trajectory was found to be on average 0.37 m/s (CI = 0.02, 1.24), residence time on average 2.2 h (CI = 0.2, 9.9, with radius r = 400 m and time threshold t = 1 h, see Methods for parameter description), and 77 % of the points were found to be of intensive movement type (MT = 1). We then investigated the relationship between the selected vertical foraging index (stBT) and each of the horizontal indices (HS, MT and RT) and assessed the potential influence of different covariates: resting while diving (RestingD), dive depth (Depth), predatory tactic (Ptactic), and movement direction with respect to haul-out sites (Direction, for a description of the covariates see Table 1 and Fig. 1). In order to do this, we fitted mixed effects models of standardized bottom time versus each of the horizontal indices and added a set of covariates, through forward model selection, with individual as a random effect. The relationship between vertical and horizontal foraging indices was not assumed to be causal but rather correlative, although we chose to model the vertical index versus the selected horizontal index (rather than the opposite) because more covariates were assumed to affect the vertical dimension and to a wider extent (see Fig. 1). Since a movement response to the intensification of search would induce an increase in standardized bottom time but a decrease in horizontal speed (HS), we used –HS as a horizontal index in order to obtain a positive relationship in case of similar response. For residence time a transformation (–1000/RT) was used to linearize the relationship with the vertical index and to scale the values for comparison with horizontal speed [see Additional file 3].

**Table 1:** Description of the variables used in the models. stBT was used as the dependent variable (vFI), HS, MT or RT as the predictors of interest (hFIs) in separate models and the remaining variables as covariates

	**Name**	**Type**	**Description**
vFI	stBT	continuous	standardized bottom time, ∈ [0, 1]
hFIs	HS	continuous	horizontal speed (m/s)
	MT	categorical	movement type, 0 = extensive, 1 = intensive movements
	RT	continuous	residence time index (h)
Covariates	Depth*	continuous	mean dive depth (m)
	RestingD*	categorical	0 = 50 % of time or less resting while diving, 1 = more than 50 % of time resting while diving
	Ptactic*	categorical	0 = 50 % or less of benthic dives, 1 = more than 50 % of benthic dives
	Direction	categorical	‘inward’ = persistent decreasing distance from a haul-out site ending within 2 km of it, ‘outward’ = persistent increasing distance from a haul-out site starting from within 2 km of it, ‘other’ = no persistent directionality and not starting or ending within 2 km from a haul-out site

*The values are summary statistics of dive characteristics per trajectory segment

**Fig. 2:**
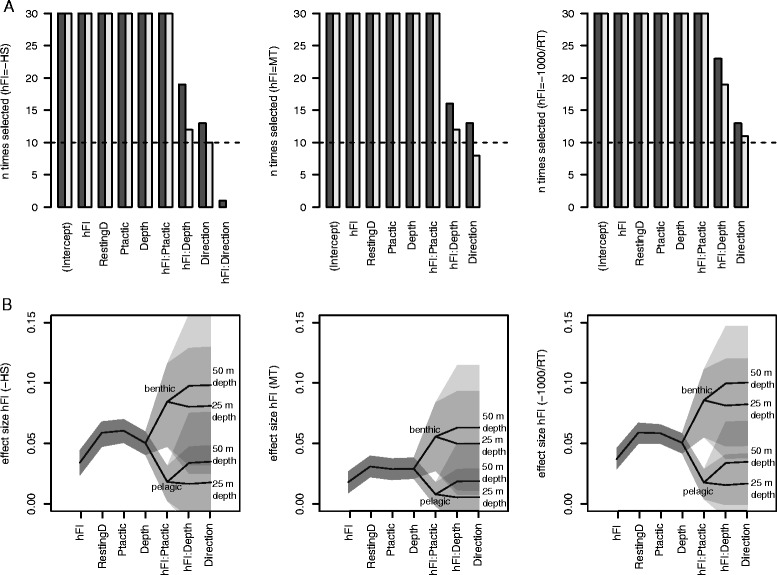
Variable selection and evolution of the hFI’s effect size in models with increasing complexity. The x axis shows the variables sequentially included in the model during forward model selection (ordered by most frequent selection order during the 30 repetitions). The final models (respectively for hFI = –HS, MT and –1000/RT, left to right columns) were chosen as the ones including all covariates selected more than a third of the times (horizontal dashed line) using a *p*-value threshold of 0.05 (black bars, **a**). The procedure was run with a *p*-value threshold of 0.01 for comparison (grey bars, **a**). The changes in the effect size of each of the hFIs with increasing model complexity is presented in (**b**), with values for the different levels of Ptactic (benthic and pelagic) and for 25 and 50 m dive depth. Grey shadings show the 95 % bootstrapped confidence intervals of the effect size

The models with the different horizontal indices (–HS, MT and –1000/RT) showed almost identical results in random structure, covariate selection, and parameter values (Fig. 2, Table 2). In all three cases the most complex random structure, including both a random intercept and slope, was selected in spite of simpler structures (see Methods) for most of the model fitting repetitions (94 % of time for –HS, 70 % for MT and 97 % for –1000/RT). This suggests that both the value of the vertical index (intercept) and its relationship with the horizontal index (hFI slope parameter) varied among individuals. The R^2^ values for the final models ranged between 31-32 % (marginal R^2^ = variance explained by fixed factors) and 35-39 % (conditional R^2^ = variance explained by fixed and random factors, see Table 2) [38].

**Table 2:** Bootstrapped parameter estimates and standard errors for the final models with the three hFIs

**Variables**	**Coefficients**		**Coefficients**		**Coefficients**	
	**(hFI = –HS)**		**(hFI = MT)**		**(hFI = –1000/RT)**	
	**Estimate**	**Std. error**	**Estimate**	**Std. error**	**Estimate**	**Std. error**
(Intercept)	0.480	0.007	0.481	0.007	0.482	0.006
hFI	0.001	0.011	–0.008	0.008	–0.001	0.009
RestingD*	–0.278	0.009	–0.273	0.009	–0.278	0.009
Ptactic*	0.138	0.007	0.080	0.008	0.139	0.006
Depth	0.002	<0.001	0.002	<0.001	0.002	<0.001
hFI:Ptactic*	0.063	0.011	0.044	0.009	0.066	0.009
Direction* (’inward’)	0.013	0.005	0.012	0.005	0.012	0.005
Direction* (’other’)	0.006	0.005	0.007	0.005	0.004	0.005
hFI:Depth	0.001	<0.001	0.001	<0.001	0.001	<0.001
	**Random variance**		**Random variance**		**Random variance**	
	**Estimate**	**Std. Error**	**Estimate**	**Std. Error**	**Estimate**	**Std. Error**
(Intercept)	0.0004	0.0002	0.0004	0.0002	0.0013	0.0003
hFI	0.0006	0.0003	0.0005	0.0003	0.0009	0.0004
Residual	0.0243	0.0004	0.0242	0.0004	0.0239	0.0004
**Marginal R** ^**2**^	0.312		0.322		0.320	
**Conditional R** ^**2**^	0.388		0.353		0.386	

*The reference level is 0 for RestingD and Ptactic, and ‘outward’ for Direction (see Table 1)

Forward model selection retained all the main covariates investigated (RestingD, Ptactic, Depth, Direction, Fig. 2A), indicating that these factors all affected the value of the foraging index in the vertical dimension (stBT). In particular RestingD and Ptactic had the strongest effect size (Table 2). Standardized bottom time, theoretically bound between 0 and 1, generally ranged between 0.22 and 0.93 (95 % CI). Its expected value for periods of resting while diving was 0.28 ± 0.008 lower than for active periods, while it was 0.14 ± 0.007 higher for periods of benthic diving than of pelagic diving (0.08 ± 0.008 for hFI = MT, Table 2), implying that harbour seals spend larger proportions of the dive duration at the dive’s bottom for active benthic dives.

For all models, the relationship between the foraging indices was always positive, and the addition of covariates to the simplest models (both as main factors and interactions) generally increased the strength of the relationship, supporting hypothesis 2 (Fig. 2b). When using –HS as a horizontal index for example, its slope for the simplest model with no covariates was found to be 0.034 (CI = 0.024, 0.044), indicating a weak relationship. The inclusion of RestingD in the model indirectly affected the slope of –HS, eliminating the confounding effect of resting while diving and increasing the slope parameter to 0.059 (CI = 0.047, 0.068). The addition of the interaction of horizontal speed with predatory tactic had the highest influence on the effect size of horizontal speed. In fact, the effect size of –HS for pelagic diving (0.001, CI = –0.024, 0.021) was found not different from zero, suggesting no relationship between the foraging indices during a pelagic predatory tactic, while the relationship was instead positive for the benthic predatory tactic (0.064, CI = 0.018, 0.103). The strength of the relationship between indices also tended to be slightly higher with depth (a 10-folds increase in depth corresponded to an increase of 0.01 ± 0.0002 in the effect size of –HS (see Table 2 and Fig. 2b).

We additionally investigated how a methodological choice, such as temporal resolution, would affect the results. We ran a sensitivity analysis by repeating the model selection procedure for decreasing temporal resolutions of the data (1, 3 and 5 h). We found that slightly simpler models were selected at lower resolutions which included the main covariates and only one interaction (hFI:Ptactic) for all horizontal indices. However, no major differences were found for the effect size of the horizontal indices [Additional file 4].

**Fig. 3:**
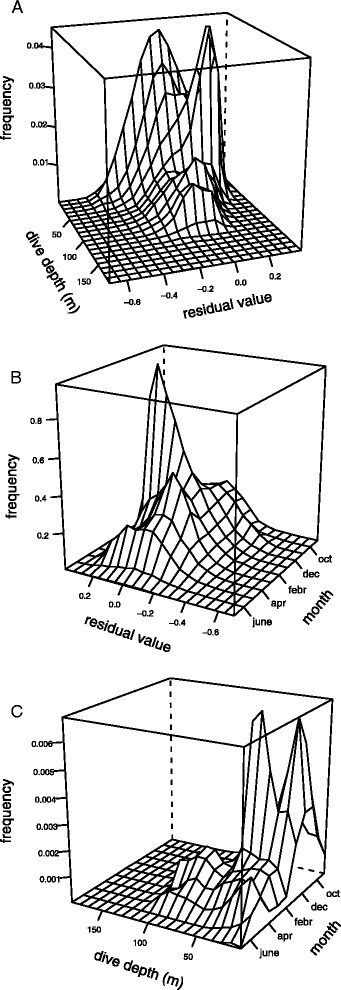
The distribution of model residuals for benthic diving, against dive depth and time. The frequency distribution of residuals from the final model with hFI = –HS plotted against dive depth (**a**) and month (**b**) showed a positively biased peak at 50 m depth around January. This also corresponds to an increased frequency of diving around 50 m during that period (**c**). Residuals for benthic diving with hFI = MT and –1000/RT showed very similar patterns and are not presented in the figure

Model validation was performed by visual examination of residuals patterns. No violation of homogeneity of variance nor normality assumption were evident and the distribution of the residuals for each individual was centred on zero, indicating that individual variation was well accounted for in the model’s random structure. However, the frequency distribution of residuals for benthic diving behaviour showed a strong positive bias along a depth axis at around 50 m, indicating harbour seals tend to spend more of the available dive time at the bottom of the dives at this depth (Fig. 3a). The positive bias was also present along a temporal axis, indicating a potential seasonal effect on the residuals (Fig. 3b). The distribution of dive depths over time however revealed that the seasonal pattern in the residuals was reflected in the seasonal pattern in dive depths, with the peak of positive residuals in January corresponding to the peak in dive depths at around 50 m in the same period (Fig. 3c). The pattern was identical for benthic residuals from the three models with the different horizontal indices (the correlation between the residuals of HS and MT models and HS and RT models were respectively 1.0 and 0.9), but was absent in residual plots for pelagic diving behaviour [Additional file 5].

## Discussion

Our results show that changes in movements, leading to an intensification of search detected by vertical and horizontal foraging indices, occur simultaneously in the two spaces, most likely as a similar behavioural response to a common cause (*i.e.* assumed increased profitability). However, the relationship between the horizontal and vertical responses has different strengths in different situations and emerges only after correcting for specific factors.

All harbour seals caught during this study were juveniles. This was most likely a consequence of the difficulty of catching more experienced and therefore older animals, even though considerable effort was put into it. Younger seals have in general lower diving capacities than adults, but harbour seals develop their diving physiology at a relatively young age, with yearlings showing values similar to adults [39]. Body size, and therefore age, is also known to affect trip durations and distances [40], but on average larger differences in the horizontal movements of harbour seals have been found between different geographical areas rather than between individual’s characteristics (*e.g.* size and sex) [41]. This suggests that our results should not be affected by the age sample and are most likely representative for the population in focus. In this study, the home range of the tagged individuals was limited to the fjord system with relatively short distances between foraging grounds and haul-out sites. Therefore a clearer distinction between extensive and intensive horizontal movements can be expected for seals foraging across wider areas.

### Predatory tactics

The major factor found to influence the relationship between the intensity of search in the horizontal and vertical spaces was predatory tactic. The value of the vertical foraging index was both found to be higher during a benthic predatory tactic and to have a stronger positive relationship with the horizontal foraging indices. The first result may be explained by the fact that the sea bottom acts as a limiting factor inducing persistence at a given depth layer, concentrating and therefore increasing the time at one depth. This may also be the reason why a clearer response in the vertical index is seen for benthic rather than pelagic diving, leading also to a stronger correlation with the horizontal indices. The lower strength of the relationship for pelagic dives may also be due to potential missed identification of part of the resting behaviour while diving. The identified resting dives were mostly pelagic (67 %) and had a strong negative correlation with the horizontal indices, failure to identify part of them would therefore weaken the relationship of the indices during pelagic active diving behaviour.

The distribution of pelagic prey fish has higher temporal and spatial variability than bottom dwelling fish [42]. Seals searching for benthic prey patches will be able to use fixed environmental cues (*e.g.* bottom topography) to find patches on subsequent trips, while pelagic prey patches will necessarily be more motile and harder to relocate. Pelagic patches will be less predictable also across the water column, since they are not bound to the sea bottom. Hence, more of the water column will be visited by the animals while searching for food, implying a more variable use of the depth layers during a dive, opposed to a simpler distinction between travelling sections (descent and ascent) and searching at the dive bottom. The comparison between benthic and pelagic dives in different penguin species has also given similar results, with benthic dives showing a longer and more efficient use of the bottom depth, while pelagic dives were described as maximizing the volume of water swept during search rather than time at a certain depth interval [43, 44]. Our results indicate that indices based on time at the bottom of dives may not be particularly robust in detecting foraging during pelagic diving, since the main variation in the index reflects the shift between resting and active diving. Other dive characteristics should therefore be investigated (*e.g* wiggles, [45]).

Many studies on pinnipeds’ diving behaviour have also shown a certain degree of association of the shapes of dive time-depth profiles with different functions and behaviours [19, 20]. Our results support the tendency of benthic dives to be more ‘squared’ (*i.e.* with steeper vertical descents and ascents, hence higher stBT) then pelagic dives, which tend to be ‘v-shaped’. However our results point out that the distinction between these shapes is mainly indicative of their location in the water column (*i.e.* benthic vs. pelagic), rather than distinguishing between travelling and foraging.

For benthic foraging, the distribution of the model residuals showed peculiar patterns along a depth gradient, with a strong positive bias at around 50 m. This may suggest a generally higher profitability at this water depth in the area studied. However, this increase in allocation of searching time occurs only during a relatively long but limited period of time (one month), suggesting the possibility of a behavioural response to a temporary but generalized decrease in resource quality or availability (*i.e.* lower mean resource quality leads to longer times spent in each foraging patch on average, marginal value theorem) [46]. Alternatively, the residual positive bias may be due to a shift in the targeted benthic resource and in the searching strategies adopted. Bowen et al. [33] have described the presence of several predatory tactics in harbour seals, which lead to different dive time budgets depending on the targeted prey type and behaviour. Very high residuals may be related to the need of a longer and more efficient use of searching time at depth, due to for example a switch to more cryptic benthic prey or to a sit-and-wait hunting strategy.

The preliminary results of scat analyses for the harbour seals’ population in the area have shown the presence in their diet of benthic prey species such as gadoids, sculpins and flatfish. This suggests a potential difference in detectability among prey species, with sculpins and flatfish being less conspicuous because hidden in the substrate. Harbour seals have been reported to use in these cases a ‘cruising’ searching tactic, scanning the sea bottom at slow swimming speed and catching multiple prey sequentially [33]. This behaviour most likely results in longer times at the bottom. For more conspicuous prey a ‘pursuit’ tactic has been described, which is most likely of shorter duration and with very different energetic implications. A shift in the targeted prey may therefore result in very different strategies of use of time at depth. At the same time, the general abundance of fish in the study area has been measured across seasons and found to be generally lowest in winter [Institute of Marine Research, unpublished data], partially supporting the conclusion of a general decrease in resource profitability.

Further investigation is therefore needed to shed light on the time and energetic budgets of underwater predatory behaviour in foraging harbour seals. This may be aided by recent advances in tag technology, such as the incorporation of accelerometry and orientation data [47, 48], which in turn will support the development of foraging indices able to accommodate a wider range of movement responses while foraging.

### Resting while diving, a confounding factor

A factor with a strong but indirect effect on the relationship between the indices was the presence of resting behaviour while diving. During this behaviour, the animals tend to increase residence in the horizontal plane and show lower standardized bottom time, leading to a negative relationship between the indices. The increase in residence in the horizontal place also leads to confusion in the distinction between foraging and resting areas from horizontal foraging indices only [36]. At the same time, resting while diving was found to be the most influential covariate explaining shifts in standardized bottom time. This indicates that considering indices of vertical searching intensity alone would allow distinguishing between periods of resting and active behaviour, rather than extensive and intensive search. Failure to account for the negative effect of at-sea resting behaviour in the relationship would decrease the measured size effect of the horizontal foraging indices by a factor of two (Fig. 2b).

### Dive depth

Dive depth, had a relatively small impact on the absolute value of the vertical indices. However, even though bottom time had already been standardized across depths by accounting for different vertical travelling times (stBT, see Methods), dive depth was still found to have a small positive effect on this variable. This indicates that harbour seals tend to be more efficient in time usage at greater depths by using on average a higher ratio of time at the bottom compared to the maximum observed for a given dive depth and duration. This is in line with the predictions of optimal diving models based on the marginal value theorem [46], where average time in patch (at the dive’s bottom) is generally expected to increase with travel time (dive depth), up to certain depths, when the animal will face oxygen limitations [21]. This can be achieved by increasing dive duration or the efficiency of use of time at depth (stBT).

### Trip direction

The main direction of the trip section with respect to haul-out site only slightly influenced the value of the vertical foraging index, indicating that the animals search with the same vertical intensity on both the outbound and inbound part of a foraging trip, and had no effect on the relationship between the foraging indices in the two spaces. This may be partially due to the fact that trip direction and horizontal indices are to some extent correlated (‘outward’ and ‘inward’ trips sections have faster speeds than ‘other’) and that trip direction did not explain any additional variation in vertical searching intensity. The different trip directions were assumed to be associated with different degrees of satiation and a lower intensity of search was expected during the returning part of the trip from the foraging grounds back to haul-out sites. The lack of explanatory power of trip direction may therefore indicate that satiation cycles do not correspond necessarily to the general need to return in the vicinity of haul-out sites. As previously noted in Ramasco et al. [36] in fact, activity cycles are often separated by resting events at sea and may occur at a smaller temporal scale than entire trips.

### Temporal resolution

Robinson et al. [12], in a study comparing different proxies of foraging in the horizontal and vertical spaces, concluded that temporal resolution and location error may have been the major cause of the low degree of agreement between measures. The present study is based on locations with a higher resolution and a smaller error (GPS vs. ARGOS locations), however the sensitivity analysis on temporal resolution (up to 5 h) did not indicate this to be an issue, despite the relatively localized movements. We therefore conclude that temporal resolution should probably not be invoked as the “default” reason for the lack of correlation between vertical and horizontal indices of foraging.

### Implications for the use and interpretation of foraging indices

**Fig. 4:**
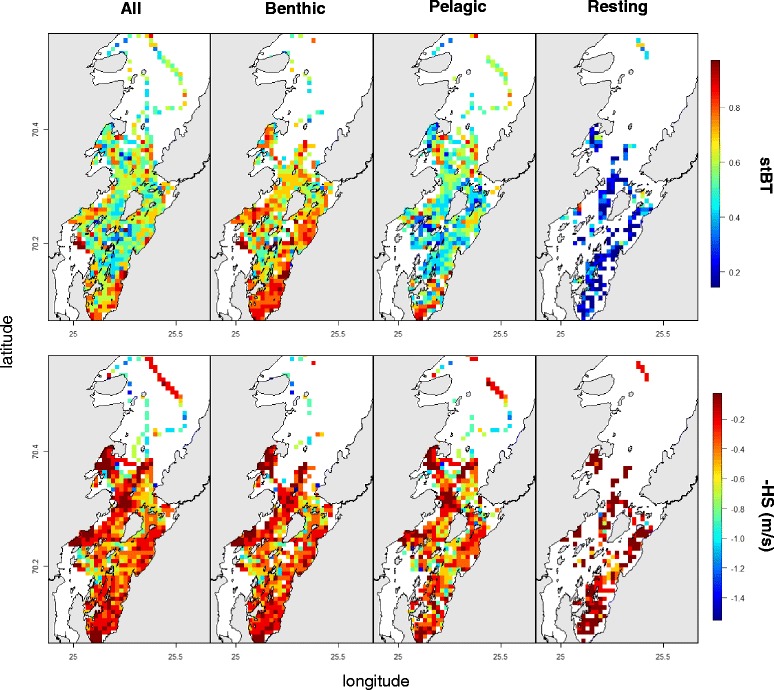
The values of hFI and vFI for different predatory tactics and resting activity. The plots show a comparison between the values of vFI = stBT (upper line) and hFI = –HS (lower line) for different behavioural states. While the first plot for each line shows the overall values across behaviours, the remaining plots show the same data divided by the most influential behaviours (active benthic diving, active pelagic diving and resting while diving). The data shown are for individual pv30-06-09 between September 2009 and March 2010 and were concentrated in a restricted area in the inner part of the fjord

The results of this study have shown that several factors affect the behavioural response of the animals while foraging, causing large variations in foraging indices (Fig. 4). Particular caution must be taken when considering changes in movements in either the horizontal plane or the vertical dimension alone. When investigating time at the bottom of dives to infer foraging behaviour, the presence of resting dives and of different predatory tactics should be taken into account. Failure to do so, in this example, would bias the detection of foraging towards the detection of active benthic diving behaviour, therefore underestimating pelagic foraging. When considering only animal movements in the horizontal plane, ignoring resting behaviour would aggregate foraging and resting activities. The latter however, seems to occur in the same locations as the former (Fig. 4), leading to a biased interpretation of the time budgets of foraging rather than their spatial location.

The spatial patterns of the vertical and horizontal indices in this study showed different degrees of spatial aggregation (Fig. 4), supporting the hypothesis of Bailleul et al. [11] that horizontal movements may respond to large scale general environmental cues, while vertical movements may respond to more localized prey presence. Moreover, the similarity of results for models with different degrees of information integration (increasing respectively for HS, MT and RT) suggests that the movement response in the vertical dimension can be explained mainly by shifts in horizontal movement speed (*i.e.* orthokinesis, [5]), and that inclusion of information on turning frequency and repetitive visits does not provide a stronger relationship with the vertical indices. It should be noted however that these effects may partially be due to the difference in resolution of the movements in the two spaces, with the vertical ones being sampled at much higher frequency. Future improvements in the resolution of horizontal movements, for example through the use of tri-axial accelerometers [47, 48], will allow assessing the presence of a movement response on the horizontal plane occurring at more local scales.

In a similar study, Bestley et al. [18] recently assessed the relationship between the probability of switching between movement types (*i.e.* resident and directional) and changes in the diving characteristics of four seal species. Their results, in accordance with ours, showed weak relationships across spaces and high variance within and across species. However, while these authors suggested the cause of this to be a simplistic interpretation of optimal foraging theory, we argue that a too simple behavioural classification (often dichotomous, travelling vs. foraging) may contribute to the lack of correlation in other species as well.

## Conclusions

The intensification of search, most likely as a movement response to increased profitability, happens simultaneously in both horizontal and vertical spaces for the harbour seal. However, behavioural factors affect the strength of the relationship and have to be taken into account for a robust interpretation of the derived foraging indices. Vertical and horizontal movements show different aspects of behaviour and the interpretation of foraging indices should be aided by additional behavioural and topographic variables (*e.g.* distance from the sea bottom, dive characteristics, *etc.*). Our results indicate that, without joint horizontal and vertical movement information, the power to infer behavioural activity is reduced and substantial errors in interpreting search intensity and local profitability may arise. For horizontal movements, resting behaviour occurred at the same locations as foraging behaviour, artificially inflating time budgets, therefore leading to potential misinterpretations on “how profitable” a given area is, rather than where it is located. When focusing only on vertical movements and on indices based on the time spent at the dive bottom, we clearly show that the foraging tactic of the animal (benthic vs. pelagic) as well as its resting behaviour need to be accounted for. Although animals in open waters are subjected to less topographic constraints, we remark that resting behaviour is likely to be equally important for them and physiological dive limits might replace topographic ones. Our study therefore contributes to the mounting evidence showing that there is no “silver bullet” for identifying searching behaviour and local profitability in marine diving animals; instead, a careful combination of the information provided by the foraging indices in both vertical and horizontal spaces is needed.

## Methods

### Data sampling

**Fig. 5:**
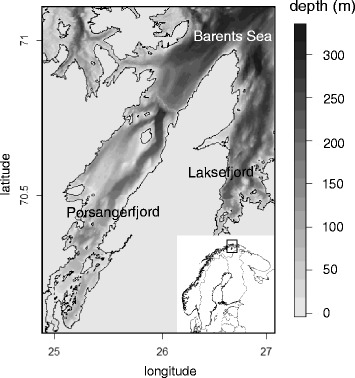
The study area and its bathymetry. The study area (main plot and rectangle in lower right plot) comprises an entire fjord system in Northern Norway: the Porsangerfjord. The fjord’s bathymetry (grey scale) ranges between very shallow areas in the inner western part of the fjord to deeper areas in the outer parts

Data on the horizontal and vertical movements of harbour seals were collected from 14 animals belonging to a resident population in the Porsangerfjord, northern Norway (70-71° N, 25-26° E, Fig. 5). The animals were equipped with GPS phone tags (SMRU Instrumentation, University of St. Andrews, UK) glued on their fur in the shoulder area right after the moulting period (July) to ensure the maximum longevity of the sampling (the tags would fall off during the next moult). Six animals were caught in September-October 2009, five in September 2010, one in August 2011 and two in September 2012. The animals’ handling procedures (capture, transport, tagging, and release) were approved by the Norwegian Animal Research Authority and are described in Ramasco et al. [36].

The tags were set to recover the animal’s GPS position at 20 min intervals. Due to the changing availability of the satellites or the tags being at times underwater, registrations were occasionally delayed, resulting in irregular time series. Time, retrieved from an onboard clock, and depth, measured through a pressure sensor, were recorded regularly at 4 sec intervals and stored in the form of time-depth profiles of 11 inflection points equally spaced in time. The maximum depth of each dive was also recorded. A conductivity sensor detected at any time if the animal was underwater or at surface. If the tag was dry for longer than 10 min, a haul-out start was registered, which ended when the tag was wet for more than 40 sec. Data were temporarily stored in the tag memory and later relayed through the GSM network.

### Horizontal movement data and foraging indices

GPS data were filtered to retain only good quality positions (maximum error of 50 m, [49]). The irregular time series of GPS positions were cut into separate bouts if no position was available for 24 h at sea or 48 h hauled out. Only the bouts of duration > 3 h were used in the analysis. Switching state-space models were fitted to the irregular locations for each individual [37]. Two states (or movement types, MT) were allowed, assumed to correspond to fast directional movements (extensive search, MT = 0) or slow and tortuous movements (intensive search, MT = 1). From the model, horizontal speed (HS) was predicted at regular 20 min intervals. Residence time (RT) was calculated from the predicted regularized locations as described in Barraquand & Benhamou [6]. The index corresponds to the time an animal spends within a circle of a given radius (r) centred on each point along the trajectory. More precisely, RT is equal to the time elapsed from the moment the animal enters the circle to the moment it leaves it for longer than a given time threshold (t). RT values within a radius (r) distance from haul-out site were excluded from these models since biased by the time of residence at the haul-out site.

### Vertical movement data and foraging indices

Errors in the registration of the seals’ vertical movements could arise due to missed surfacing registrations caused by failure of the pressure or conductivity sensors to detect respectively shallow depths or dry conditions. If one or more surfacings were missed, multiple dives were compressed into one single 11-points profile causing implausibly long dive durations at the limit of the tag’s registration capacity (25 min) and largely above expected maximum dive durations for the species (ca. 10 min, [50]). Potentially incorrect dive records were defined as the ones that, excluding the first descending and last ascending phases, showed one or more depth readings in the upper 25 % of the dive without surfacing or when the animal stayed in the upper 25 % of the maximum depth for more than 50 % of the dive duration. These thresholds were chosen assuming that an animal is neither likely to decide to dive again after reaching the upper part (upper 25 %) of the water column without surfacing, nor prone to spend over half the time submerged right below the surface without emerging before or after a deeper dive. Ninety percent of the dive records with durations at the upper edge of the distribution, but not detected by the method previously explained, were less than 5.6 m deep. This indicated that, most likely, for dives shallower than that threshold, surfacings were often missed by the sensors. Dives shallower than 5.6 m were therefore excluded from further analysis.

Bottom time (BT) was computed as the time in the bottom phase of each dive. We used the definition of bottom phase as in Austin et al. [17] as the time spent in the lower 15 % of the dive’s maximum depth. BT was then standardized across depths (stBT) by transforming it into a % of maximum potential BT (maxBT) for a given dive depth and duration. For dive i:

1 $$ \mathrm{s}\mathrm{t}\mathrm{B}{\mathrm{T}}_{\mathrm{i}}\kern0.5em =\kern0.5em \mathrm{B}{\mathrm{T}}_{\mathrm{i}}/\mathrm{maxB}{\mathrm{T}}_{\mathrm{i}} $$

2 $$ \mathrm{maxB}{\mathrm{T}}_{\mathrm{i}}\kern0.5em =\kern0.5em \mathrm{dive}\kern0.5em \mathrm{duratio}{\mathrm{n}}_{\mathrm{i}}\kern0.5em -\mathrm{minimum}\kern0.5em \mathrm{t}\mathrm{ravel}\kern0.5em \mathrm{t}\mathrm{i}\mathrm{m}{\mathrm{e}}_{\mathrm{i}} $$

Minimum travel time was defined as the time the animal would use to reach the depth of the bottom phase (15 % of maximum depth) from surface at maximum vertical speed (set as the 0.95 quantile of the individual’s distribution of vertical speeds, mean 1.97 m/s, range 1.75 – 2.16 m/s across individuals). For comparison, the Time at Depth index (TAD, [29]) was also calculated and the correlation between BT, stBT, and TAD investigated [see Additional file 2]. Since in general several dives occur between two successive locations (*i.e.* a trajectory segment), dive characteristics were averaged for each trajectory segment.

### Covariates

Among the factors potentially affecting the relationship between horizontal and vertical foraging indices, we considered the following variables, potentially affecting the searching intensity of the animals while foraging: dive depth, trip direction, and predatory tactic (benthic or pelagic). Moreover, we considered the presence of resting behaviour while diving as a potential confounding signal for the detection of foraging using indices based on the allocation of time in space (see Table 1 for a description of the covariates).

**Fig. 6:**
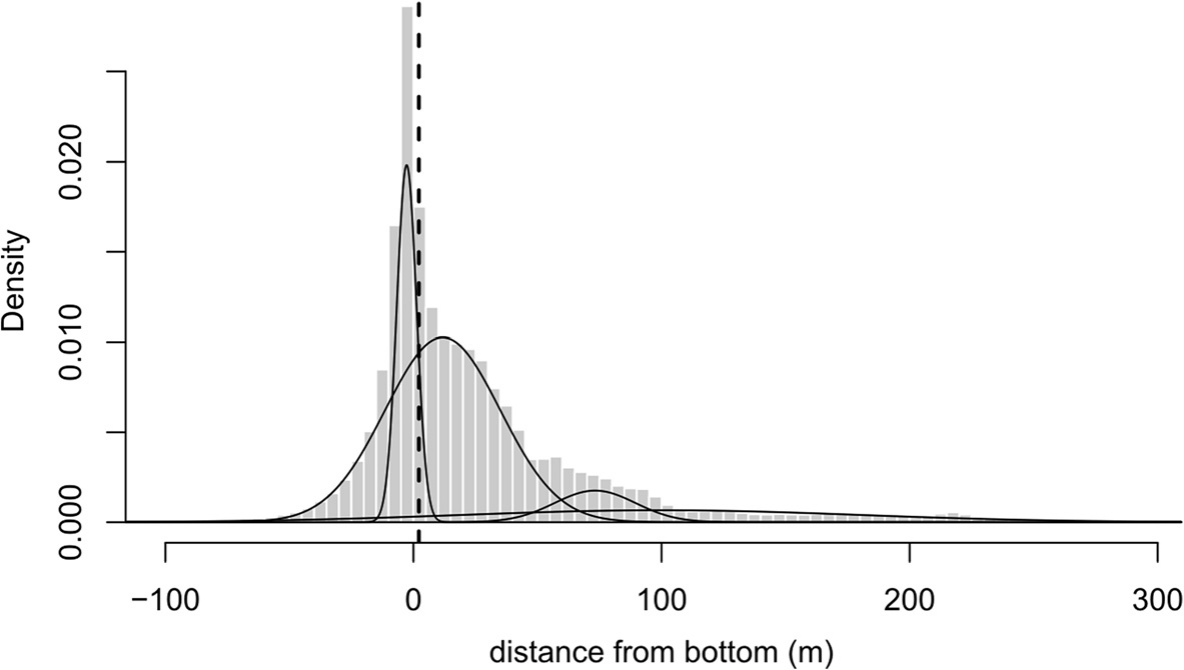
Detection of benthic and pelagic diving. The histogram shows the empirical distribution of the distance between maximum dive depth and sea bottom, while the lines show the fitted mixture of *n* normal distributions (best model for *n* = 4). The fitted distribution with mean closer to 0 was assumed to be the distribution of benthic dives (mean – 3.15 m). The dashed line shows the threshold (2.2 m) used for distinguishing between benthic (<= 2.2 m) and pelagic dives (>2.2 m)

To differentiate between periods of benthic and pelagic diving behaviour, we calculated for each dive > 5.6 m the distance between its maximum depth and the depth of the sea bottom, expressed as the depth of the water column at mid tide (modelled bathymetry, grid cell 100 × 100 m, data from the Norwegian Mapping Authority [51]). Each dive was located on the bathymetric map by assuming constant swim speed on a straight line between two successive GPS locations. To account for the variance in the estimated distance to the sea bottom, which is due to the combined errors in dive location, bathymetric predictions and tidal state, we fitted a mixture of *n* normal distribution functions (1 < = *n* < =5) to the frequency distribution of bottom distances and modelled the probability of each dive to belong to any of these distributions. From the best model (*n* = 4, Fig. 6) we assumed the distribution having its mean closest to zero to be the distribution of bottom distances for benthic dives. The mean and upper (95 %) quantile of that distribution were found to be respectively –2.8 m and 2.2 m, therefore all dives for which bottom distance was < = 2.2 were considered benthic. The negative average distance from the sea bottom for benthic dives suggests that the tagged individuals were diving on average in deeper waters than predicted. This bias, as mentioned above, was partially due to errors in locating dives and in the bathymetry predictions. However, given bottom depths were calculated for mid tide, the negative mean distance from the bottom can partially indicate that seals were diving more often during high tide than low tide (tidal range ±1 m).

**Fig. 7:**
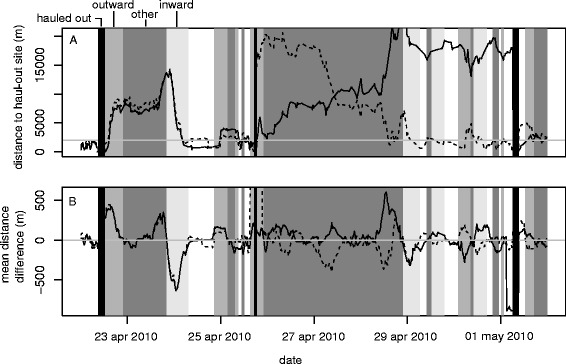
Trip direction. The categorical variable (Direction) was defined as the major movement direction with respect to the previous and next haul-out sites (black vertical bands). The distance from the last and to the next haul-out sites (respectively solid and dashed line) was calculated for each trajectory location (**a**). The difference between successive distances was then computed and averaged across a running window of 6 h (**b**). Trip segment limits were defined when average difference distance crossed zero (grey line in **b**). ‘Outward’ (medium dark grey bands) and ‘inward’ (light grey bands) trip segments were identified as the segments respectively starting and ending within 2 km from the last or next haul-out site (grey line in **a**). The Direction category ‘Other’ (dark grey bands) was identified by exclusion (see Methods). The approach used was able to identify multiple return trips to the vicinity of haul-out sites between two haul-out periods. Data shown are for individual pv30-01-09 during a selected period of time

A categorical variable (Direction) was created to reflect the major movement direction with respect to haul-out sites in each foraging trip. The different categories distinguished between movements directed away from or towards the haul-out sites, movements close to a haul-out, movements of transition between haul-out sites, or movements with no particular directionality. To classify the different parts of a trip, first the difference in distance to haul-out site was calculated for each position in a movement trajectory, then a running average was computed (window width 6 h) to smooth the fine scale variation in direction changes (Fig. 7a), and then each trip divided into sections whenever average distance difference changed sign (Fig. 7B). ‘Outward’ (and ‘inward’) trip sections were defined as the continuous succession of positions with a persistent increasing (decreasing) distance from the last (to the next) haul-out site and starting (ending) within 2 km from the haul-out site [see Additional file 6 for the choice of window width and threshold distance parameters]. Sections entirely within the threshold distance from haul-out sites (2 km) were classified as ‘within range’. Segments starting within the range of one haul-out site and ending within the next were classified as ‘transiting’. The remaining segments, showing relatively stationary movement behaviour away from haul-out sites, were by exclusion classified as ‘other’. The approach used was able to detect multiple return trips between two successive haul-out events (Fig. 7). We assumed the main trip directions to reflect the satiation state of the animals, with a higher degree of satiation during ‘inward’ trip sections rather than ‘outward’ and ‘others’. This assumption was based on the biology of the species, a short range forager and generally performing foraging trips relatively close to haul-out sites [35], allowing to assume that the individuals will on average return to the haul-out site for resting purposes after foraging [34]. The categories ‘within range’ and ‘transiting’ (respectively 21 and 4 % of points) were not considered to be related to a particular satiation state or to foraging behaviour in general and were therefore not included in the analysis.

Finally we detected periods of resting while diving as described in Ramasco et al. [36]. We calculated the proportion of vertical ascent to descent speed and termed this proportion dive skewness (SK). We assumed that periods of resting while diving would be indicated by series of consecutive skewed dives and that changes in SK would occur abruptly at the shifts between resting and other behaviours. We then used a multiple changepoint method [52] to detect breaks in continuous series of log(SK) based on shifts in the mean. The segments obtained were then classified by fitting a mixture of normal distributions to the frequency distribution of mean log(SK) for each segment (see [36] for details). We then summarized the information in a categorical variable (RestingD) indicating if the majority of the time per trajectory segment was spent resting or in activity.

### Model fitting

Trajectory segments (duration 20 min) including haul-out, surfacing or shallow diving (<5.6. m) behaviour for more than 50 % of the time were excluded from the dataset, together with segments occurring within a RT radius distance from haul-out site or belonging to the ‘transiting’ and ‘within range’ Direction categories (59 % of data). A certain degree of autocorrelation was still assumed to be present in the reduced dataset (N = 73 629), therefore resampling of *s* random subsets of *n* data points each was used to repetitively fit the models at each stage of model selection in order to reduce the effect of autocorrelation on parameter estimation.

Linear mixed effects models were fitted with individual as a random effect. Sex was not accounted for in the models, since no differences were found in vertical and horizontal indices across sexes (stBT: females CI = 0.21 – 0.94, males CI = 0.21 – 0.91; HS: females CI = 0.01 – 1.13 m/s, males CI = 0.01 – 1.20 m/s). To render the relationship between the vertical and the horizontal foraging indices linear and positive in case of increase in searching intensity in the two spaces, HS and RT were transformed into –HS and –1000/RT respectively (corr (–HS, –1000/RT) = 0.87), where the factor 1000 was used to scale their values to similar ranges for comparison of the model parameters [see Additional file 3]. For model selection, first the appropriate random variance structure was investigated by comparing three full models with respectively no random variance, random intercept (${u}_1$) and random intercept and slope for the hFI (${u}_1+{u}_2hFI$):

3 $$ vF{I}_{ij}\sim \boldsymbol{\upbeta} {\mathbf{X}}_{ij}\kern0.5em +{e}_{ij}, $$

4 $$ vF{I}_{ij}\sim \boldsymbol{\upbeta} {\mathbf{X}}_{ij}\kern0.5em +{u}_{i1}\kern0.5em +\kern0.5em {e}_{ij}, $$

5 $$ vF{I}_{ij}\sim \boldsymbol{\upbeta} {\mathbf{X}}_{ij}\kern0.5em +{u}_{i1}+\kern0.5em {u}_{i 2}hF{I}_{ij}+\kern0.5em {e}_{ij}, $$

with **βX**_*ij*_ being the matrix of covariates and their parameters, and *e*_*ij*_ the error for the ith individual and jth point. The three models, all fitted using reduced maximum likelihood estimation (REML), were compared by likelihood ratio tests (significant *p*-values < 0.01, *s* = 100, *n* = 7000) and the best structure was considered to be the one selected most often across the *s* repetitions.

Using the chosen random structure, fixed effects were then added by forward model selection, from a minimum model including only the hFI,

6 $$ vFI\sim a\kern0.5em +\kern0.5em {\beta}_1\kern0.5em hFI, $$

up to a full model including all covariates and 2-ways interactions (fitted using maximum likelihood estimation, ML),

7 $$ \begin{array}{l}vFI\sim a\kern0.5em +\kern0.5em {\beta}_1hFI\kern0.5em +\kern0.5em {\beta}_2\mathrm{R}\mathrm{e} stingD\kern0.5em +\kern0.5em {\beta}_3 Depth\kern0.5em +\kern0.5em {\beta}_4 Ptactic\kern0.5em +\\ {}{\beta}_5 Direction\kern0.5em +\kern0.5em {\beta}_6hFI*\kern0.5em  Depth+\kern0.5em {\beta}_7hFI* Ptactic+{\beta}_8hFI* Direction.\end{array} $$

The interaction between RestingD and hFI was not tested since resting dives occur almost exclusively when the animal is stationary (small values of HS, large values of RT and MT = 1). The variables were sequentially added (based on the Akaike’s Information Criterion scores) and kept in the model if a likelihood ratio test was found significant with a *p*-value threshold of 0.05 (a *p*-value threshold of 0.01 was also used for comparison, see Fig. 2). Models were fitted 30 times (*n* = 7000) and the frequency and order of selection of each covariate used to choose the best set of fixed effects. Covariates included in the models at least 1/3 of the times were chosen. The final model was fitted (using REML) and parameter errors estimated by bootstrapping (100 repetitions, *n* = 7000). Model validation was performed by visually assessing the presence in the residuals of non linear patterns or violation of the assumptions of homogeneity and normality.

To assess the influence of the temporal resolution of the data we resampled the trajectories every *p* points, with *p* = 3, 9, and 15, simulating decreasing temporal resolutions of respectively one point every 1, 3, and 5 hours (20 min * *p*). Numerical covariates were re-estimated either by averaging the values every *p* trajectory segments (for the dive variables) or re-estimating the variables from the new trajectories (for HS, RT and Direction). To avoid fitting new switching state-space models at lower resolution, due to the high computational effort required for these models, movement type was estimated by assigning to the new trajectory segments the most frequent of the two states computed at the highest resolution (hence the choice of a set of uneven *p* values in order to always have a majority of either state). We then performed forward model selection and parameter estimation as previously described.

All data processing and analyses were performed in R 3.1.1 [53]. State space models were run using the *bsam* package [54]. RT was computed using the *adehabitatLT* package [55]. Mixed models were fitted using the *nlme* package [56].

## Availability of supporting data

The data set supporting the results of this article is available in the Movebank repository (ID 72527011).

## Additional files

Additional file 1: **Tagging information and sample size per individual.**

Additional file 2: **The relationship between the selected vFI and two comparable indices not used in the analysis.** The plots show the relationship between the used index stBT (mean standardized bottom time per trajectory segment) and two comparable indices: BT (mean bottom time per trajectory segment) and TAD (mean Time At Depth index per trajectory segment, [29]). A locally weighted smoothing curve (LOESS, local polynomial regression, black line) shows the trend of the relationships: positive but non-linear with BT; positive and fairly linear with TAD (linear correlation = 0.77).

Additional file 3: **The relationship between HS (horizontal speed), RT (residence time index) and its transformation (1000/RT).**

Additional file 4: **Changes in the effect size of the hFIs for decreasing temporal resolutions (20 min, 1 h, 3 h, 5 h).** The grey lines show the hFIs effect size and the grey bands the respective 95 % confidence intervals for models with different temporal resolutions (for hFI = –HS, MT and –1000/RT from left to right). The models at all resolutions include the interaction hFI:Ptactic. Their effect size is therefore shown both for benthic (dark grey line & band) and pelagic (light grey line and band) diving. The model for resolution = 20 min includes additionally the interaction hFI:Depth. The effect size in that case is shown for mean values of Depth (=30 m).

Additional file 5: **The distribution of model residuals for pelagic diving, against dive depth and time.** The frequency distribution of residuals from the final model with hFI = –HS plotted against dive depth (**a**) and month (**b**) showed normally distributed residuals. The distribution of dive depths was also centred on shallow depths except in spring (**c**). Residuals for benthic diving with hFI = MT and hFI = –1000/RT showed very similar patterns and are not presented in the figure.

Additional file 6: **Percentage of points in the Direction categories’outward’ (left plot) and’inward’ (right plot) using different averaging window widths (x axis) and thresholds of distance from** **haul-out** **site (line type, see Methods).** The chosen parameter combination (6 hours, 2 km, black circle) balances the need to maximize the number of points in the categories relevant for this study (‘outward’ and ‘inward’), while avoiding to smooth the temporal patterns excessively (the chosen window width is at the start of the plateau of the curve, where the increment in number of points with increasing width is small).

## Abbreviations

ARS: Area restricted search
BT: Bottom time
CI: 95 % Confidence interval
FI: Foraging index
GPS: Global positioning system
GSM: Global system for mobile communications
hFI: horizontal FI
HS: Horizontal speed
MT: Movement type
Ptactic: Predatory tactic, see Table 1
RestingD: Resting dives, see Table 1
RT: Residence time
stBT: Standardized BT
TAD: Time at depth index
vFI: vertical FI
