# Supplementary figures and images for: The intensity of horizontal and vertical search in a diving forager: the harbour seal
Source: Mov Ecol. 2015 May 27;3(1):15. doi: 10.1186/s40462-015-0042-9 (PMC4445568; doi:10.1186/s40462-015-0042-9)

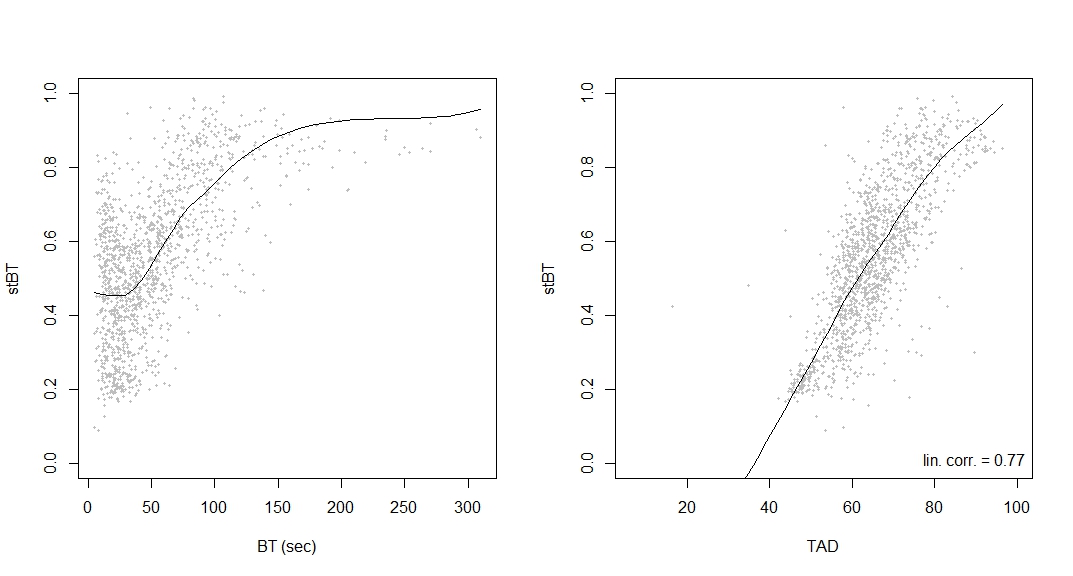

Supplement: Supplementary file 2 — The relationship between the selected vFI and two comparable indices not used in the analysis. The plots show the relationship between the used index stBT (mean standardized bottom time per trajectory segment) and two comparable indices: BT (mean bottom time per trajectory segment) and TAD (mean Time At Depth index per trajectory segment, [29]). A locally weighted smoothing curve (LOESS, local polynomial regression, black line) shows the trend of the relationships: positive but non-linear with BT; positive and fairly linear with TAD (linear correlation = 0.77). [file 40462_2015_42_MOESM2_ESM.jpeg]

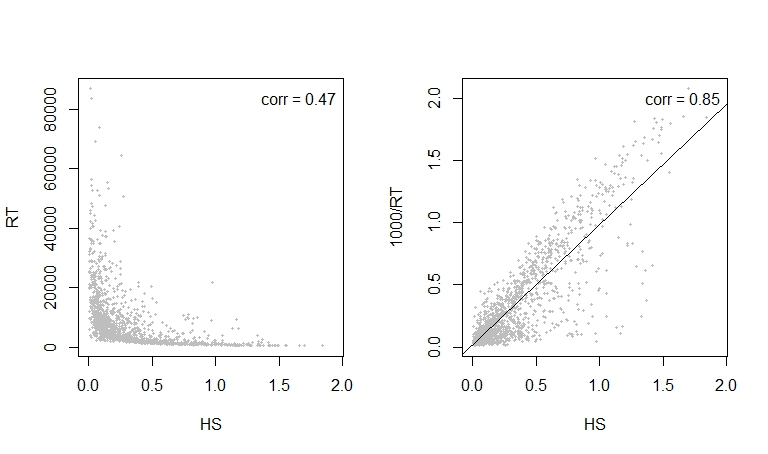

Supplement: Supplementary file 3 — The relationship between HS (horizontal speed), RT (residence time index) and its transformation (1000/RT). [file 40462_2015_42_MOESM3_ESM.jpeg]

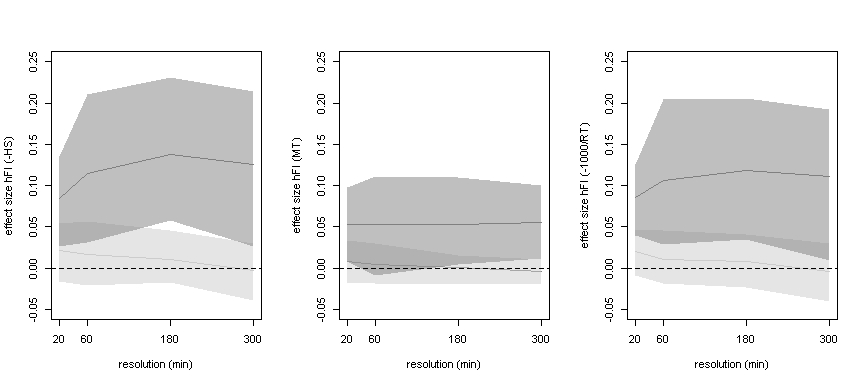

Supplement: Supplementary file 4 — Changes in the effect size of the hFIs for decreasing temporal resolutions (20 min, 1 h, 3 h, 5 h). The grey lines show the hFIs effect size and the grey bands the respective 95 % confidence intervals for models with different temporal resolutions (for hFI = –HS, MT and –1000/RT from left to right). The models at all resolutions include the interaction hFI:Ptactic. Their effect size is therefore shown both for benthic (dark grey line & band) and pelagic (light grey line and band) diving. The model for resolution = 20 min includes additionally the interaction hFI:Depth. The effect size in that case is shown for mean values of Depth (=30 m). [file 40462_2015_42_MOESM4_ESM.jpeg]

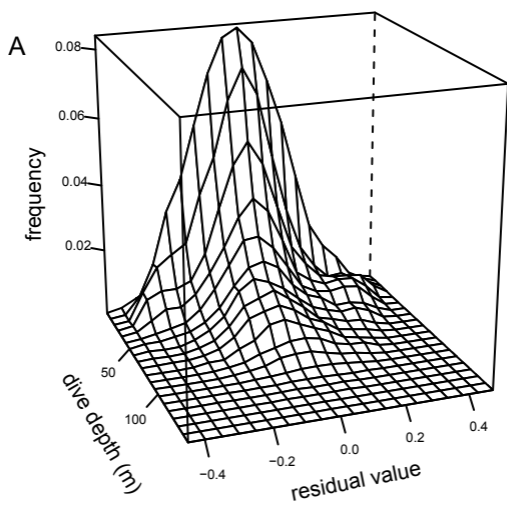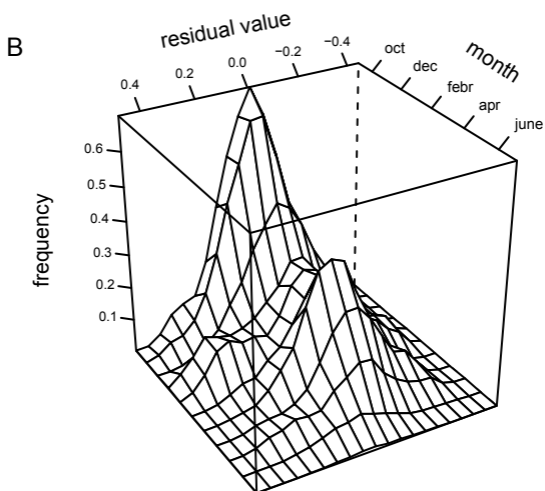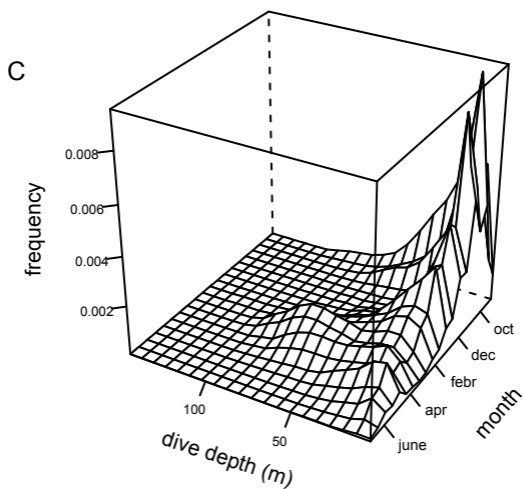

Supplement: Supplementary file 5 — The distribution of model residuals for pelagic diving, against dive depth and time. The frequency distribution of residuals from the final model with hFI = –HS plotted against dive depth (a) and month (b) showed normally distributed residuals. The distribution of dive depths was also centred on shallow depths except in spring (c). Residuals for benthic diving with hFI = MT and hFI = –1000/RT showed very similar patterns and are not presented in the figure. [file 40462_2015_42_MOESM5_ESM.pdf]

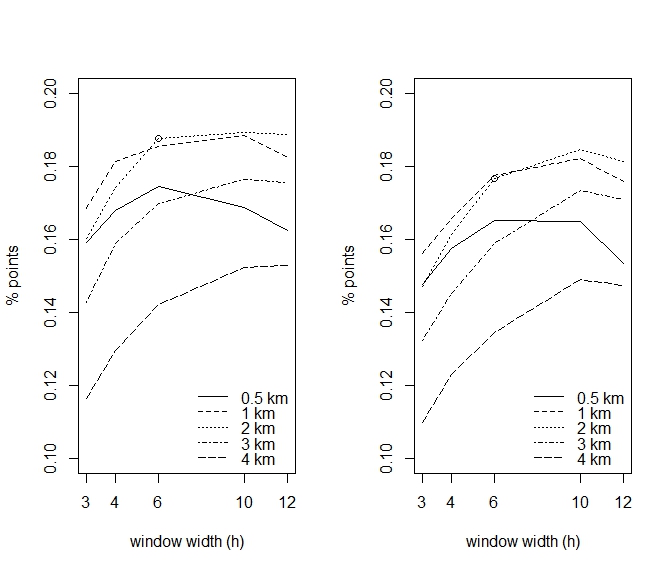

Supplement: Supplementary file 6 — Percentage of points in the Direction categories’outward’ (left plot) and’inward’ (right plot) using different averaging window widths (x axis) and thresholds of distance from haul-out site (line type, see Methods). The chosen parameter combination (6 hours, 2 km, black circle) balances the need to maximize the number of points in the categories relevant for this study (‘outward’ and ‘inward’), while avoiding to smooth the temporal patterns excessively (the chosen window width is at the start of the plateau of the curve, where the increment in number of points with increasing width is small). [file 40462_2015_42_MOESM6_ESM.jpeg]
